# Asymptomatic Bacteriuria in Kidney Transplant Recipients—A Narrative Review

**DOI:** 10.3390/medicina59020198

**Published:** 2023-01-19

**Authors:** Justyna E. Gołębiewska, Beata Krawczyk, Magdalena Wysocka, Aleksandra Dudziak, Alicja Dębska-Ślizień

**Affiliations:** 1 Department of Nephrology, Transplantology and Internal Medicine, Medical University of Gdańsk, 80-210 Gdańsk, Poland; 2 Department of Molecular Biotechnology and Microbiology, Faculty of Chemistry, Gdańsk University of Technology, 80-233 Gdańsk, Poland; 3 Digital Experimental Cancer Medicine Team, Cancer Biomarker Centre, CRUK Manchester Institute, University of Manchester, Manchester M13 9PL, UK; 4 Microbiology Laboratory, University Clinical Center, 80-952 Gdańsk, Poland

**Keywords:** asymptomatic bacteriuria, kidney transplantation

## Abstract

Urinary tract infections (UTIs) are the most prevalent complications in kidney transplant (KTx) recipients. The most frequent finding in this group of patients is asymptomatic bacteriuria (ASB). Here, we provide an overview of the available evidence regarding ASB in KTx recipients, including its etiopathology, clinical impact and management. There is a growing body of evidence from clinical trials that screening for and treating ASB is not beneficial in most KTx recipients. However, there are insufficient data to recommend or discourage the use of a “screen-and-treat strategy” for ASB during the first 1–2 months post-transplant or in the case of an indwelling urinary catheter. Despite its frequency, ASB after KTx is still an understudied phenomenon.

## 1. Introduction

Urinary tract infections (UTIs) are the most prevalent complications in kidney transplant (KTx) recipients [1,2,3,4,5]. The clinical manifestation or phenotype of symptomatic infections may vary from cystitis to urosepsis. However, a frequent finding in this group of patients is asymptomatic bacteriuria (ASB) [6,7,8,9], which is nowadays considered to be more a form of colonization than infection. The reported prevalence of ASB varies greatly depending on the study, population enrolled, time elapsed since KTx, use of antibiotic prophylaxis and length of follow-up from 3% to 50% [8,10]. ASB is the most frequent clinical phenotype in the first month after KTx, after which symptomatic infections predominate and the relative proportion of ASB decreases [8,11].

## 2. Definitions

As stated in the guidelines of the Infectious Diseases Society of America (IDSA), ASB is defined in patients without indwelling catheters as the presence of ≥10^5^ colony-forming units (CFU)/mL (≥10^8^ CFU/L) in a voided urine specimen, without coinciding signs or symptoms typical for UTI [12,13,14,15]. For men, a single urine specimen is sufficient for diagnosis, while in women two consecutive specimens should be obtained to confirm the persistence of bacteriuria [16]. This is because in up to 60% of women bacteriuria is transient, and is not present on repeat screening after an initial positive specimen [17,18]. In patients with indwelling devices, the presence of ≥ 10^5^ CFU/mL is still the best diagnostic criterion for bladder bacteriuria [19]. Lower quantitative counts of bacteria (≥10^2^ to <10^5^ CFU/mL) are likely to be a contamination of the urine specimen from the biofilm covering the device rather than true bacteriuria. The presence of from ≥10^2^ to <10^5^ CFU/mL of bacteria in urine specimens collected using “in and out” catheterization or following the placement of a new indwelling catheter points to true bacteriuria, but these lower quantitative counts have undetermined clinical significance in asymptomatic patients. The above listed definitions are true when traditional culture-based methods are used to assess if bacteria are present in the urinary tract. Recent studies using metagenomics have shown that urine is not sterile, as was previously believed, but even in healthy subjects a community of microorganisms termed the microbiome is present [20,21].

## 3. Etiopathology

Due to the abundance of virulence factors, uropathogenic strains adapted to various ecological niches have acquired the ability to evade the host’s immune response [22]. Polysaccharide capsules, fimbrial and afimbrial adhesins, the iron uptake system (siderophores) and lipopolysaccharides (LPS) are some of the important virulence factors that facilitate bacterial survival and development of infection.

The relationship between UTI and ASB is not well understood. It is possible that the absence of UTI symptoms in the presence of bacteria may follow colonization of the urinary mucosa by defective strains that are unable to induce a full-blown inflammatory response. *E. coli,* which is the most prevalent uropathogenic strain, has been most extensively studied in this context. However, there is no rigorous molecular definition to differentiate between uropathogenic or merely urocolonizing strains. *E. coli* strains causing symptomatic infections and ASB showed different virulence gene repertoires. The UTI isolates were more likely to have the papEF and fyuA genes, while the feoB gene was significantly less common than in ASB isolates. The cluster of the virulence gene fimH (+) kpsMTII (+) feoB (+) was significantly more common in ASB strains. Strains causing ASB were also more resistant to a significant proportion of the antimicrobials tested [23]. Rice et al. found that ASB progression to symptomatic infection in KTx recipients was associated with a unique pattern of adherence factors with the expression of P fimbriae but not Dr fimbriae [24]. This finding was confirmed in a prospective study using bacterial whole-genome sequencing to analyze 72 *E. coli* strains isolated from urine of 54 KTx recipients. In this study, the prevalence of pap genes, i.e., the number of genes required for the production of P fimbriae was significantly higher in *E. coli* isolates from cases of acute graft pyelonephritis (AGPN) than in cases of ASB [25].

According to Salvador et al., *E. coli* ASB strains have a reduced virulence potential due to the loss of virulence genes or a lack of expression of genes encoding them. It is likely that long-term colonization of the urinary bladder, especially in hospitalized patients, leads to attenuation of the virulence phenotype as a result of adaptation to the host environment [26]. It was observed that negative expression of fimbriae type 1 and fimbriae type P was a result of partial deletions of the encoding determinants. The authors established that the dominant phylogenetic lineage of ASB was clonal complex 73 (CC73). However, another analyzed line, ST95, was less frequently included in ASB isolates [26]. Some investigators have also reported biofilm formation to be higher in ASB strains [27,28,29,30], but this phenomenon was later questioned [18]. Eberly et al. performed large-scale metabolomic analysis of 300 ASB- and cystitis-associated isolates to find a distinct metabolic signature, especially one related to purine metabolism, differentiating cystitis strains from ASB strains [31].

Gołębiewska et al., who investigated gene profiles encoding virulence factors of *K. pneumoniae* isolated from KTx recipients with ASB, demonstrated that in ASB isolates the kpn gene encoding FimH-like adhesin was significantly more prevalent as compared with isolates causing symptomatic UTIs. It was shown that the kpn gene may replace the expression of type 1 fimbriae, promoting bacterial colonization and endurance in ASB cases [32].

The host–pathogen interaction in cases of ASB involves not only changes in the expression of bacterial functional virulence factors and metabolism, but also the ability to modulate the host’s immune response to allow the survival and persistence of asymptomatic strains, e.g., by reducing the expression of the host’s immunity-related genes [33]. In another work, *K. pneumoniae* NDM-1 strains were isolated from KTx recipients, some of them suffered due to UTI and others had ASB or were only colonized. The genetic background of bacteria was examined using WGS (whole genome sequencing). It was found that the strains had high genetic similarity and clonal origin. The profiles of virulence were similar too, regardless of whether they caused only colonization, ASB or UTI. The authors suggested that, for some patients, the innate immune response may not be strong enough to generate symptoms [34]. Asymptomatic colonization of the human bladder by ASB *E. coli* strain 83,972 has been shown to suppress selected disease-associated signaling pathways [35,36]. Heitmuller et al., using model insect host *G. mellonella* larvae infected with either uropathogenic or urocolonizing *E. coli* strains, showed strain-specific differences in the class and expression levels of genes encoding antimicrobial peptides, cytokines and enzymes controlling DNA methylation and histone acetylation [37]. Even though ASB strains are able to prevent the development of a systemic inflammatory reaction, they may still induce a local inflammatory response. In patients with low-grade, papillary, and non-muscle-invasive tumors and chronic ASB the number of recurrences and recurrent tumors was lower, and tumor-free survival times were longer than similarly staged uninfected patients. Therefore, it has been hypothesized that urocolonizing strains may reduce bladder tumor recurrence by activating local immune mechanisms [38]. In KTx recipients, the urine IL-8 level was elevated in both patients with symptomatic UTI and in cases of asymptomatic bacteriuria in comparison to the control group, most probably indicating an ongoing occult inflammatory process in the urinary tract [39].

## 4. Predisposing Factors

Many pre-, peri- and post-transplant factors are believed to increase the risk of UTIs, including ASB, in KTx recipients. These include female gender, diabetes mellitus, immunosuppression and kidney allograft dysfunction or rejection, underlying urinary tract abnormalities and instrumentation of the urinary tract, including ureteral stent placement and prolonged urinary catheterization. So far, the dose or type of maintenance immunosuppression has not been found to impact the risk of UTIs. Various authors have suggested different potential ASB risk factors. The only consistent finding was the increased risk of ASB in females [8,10,40]. In a study by Coussement et al., female KTx recipients over the age of 50 were the population at highest risk of having ASB [10]. In an analysis of 2363 UTI events recorded in 2368 KTx recipients, including 1111 episodes of ASB, the 1-year incidence of ASB was also significantly higher in females [11]. Notably, an association between the Charlson Comorbidity Index (CCI) [41] and risk of ASB was suggested in one of the studies. While the authors did not find a statistically significant association between risk of ASB and any specific comorbid conditions, they showed that upon multivariate analysis CCI that takes into account a variety of medical conditions, how advanced they are, together with age, also predicting long-term mortality, was a predictor of ASB. [8].

## 5. Etiology

**Figure 1:**
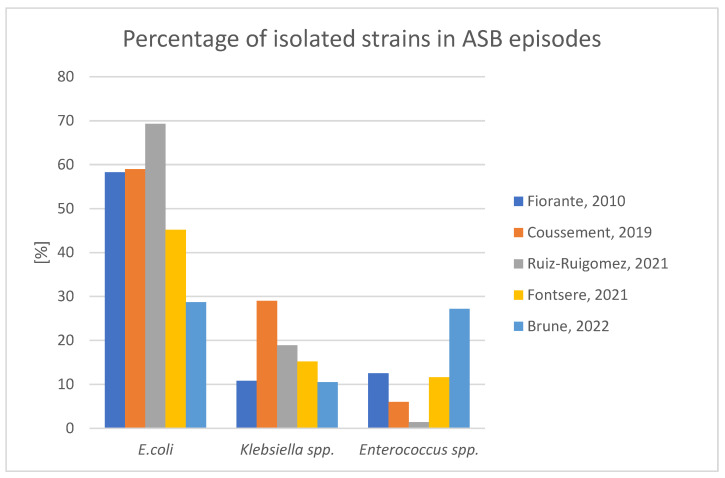
The most common bacteria isolated in asymptomatic bacteriuria episodes according to various authors.

The etiology of ASB mirrors the etiology of symptomatic UTIs, with a predominance of Gram-negative rods typically colonizing the gastrointestinal tract such as *E.coli* or *Klebsiella* spp. It is noteworthy that strains isolated early post-KTx differ from those found in late periods after KTx. In a single center study by Gołębiewska et al., in the first month after KTx *E. faecium* predominated, followed by *E. faecalis* and *E. coli*. Beginning from the second month, *E. coli* was the most frequently isolated urinary pathogen, followed by *Proteus* species and *Klebsiella* species [8]. Brune et al., in a multicenter observational study, reported distinct pathogen profiles in ASB compared to UTIs, including urosepsis. Specifically, in ASB the proportion of *Enterococcus* species and coagulase-negative *Staphylococci* was significantly higher than in symptomatic infections. The pathogen profile in symptomatic infections included *E. coli*, *Enterococcus* species and *Klebsiella* species, which accounted for about 85% of all causative agents. Notably, the pathogen profiles in all UTI phenotypes remained stable over the first twelve months after KTx and were not affected by either the removal of the double J-stent or the use of prophylaxis with trimethoprim-sulfametoxazole [11]. The frequency of the three most common etiological agents in ASB episodes according to various authors is presented in Figure 1.

## 6. Clinical Impact of ASB

At least in theory avoiding UTI in KTx recipient might improve patient and kidney allograft survival. In KTx recipients microorganisms colonizing the lower urinary tract and causing ASB may constitute a hazard for upper urinary tract invasion and development of full-blown symptomatic infection, including urosepsis. In the setting of immunosuppressive therapy, the host’s immune response may not offer a chance for a spontaneous recovery from even a benign infection, especially soon after KTx. The available data on the effect that UTIs have on long-term kidney allograft function are inconclusive. The true influence of various phenotypes of UTIs on both the recipient and kidney allograft outcome has so far not been established. The general belief is that asymptomatic bacteriuria (AB) is benign, in contrast to symptomatic upper UTIs, such as acute graft pyelonephritis or urosepsis. However, even if UTIs do not affect kidney allograft survival directly, they can pose a significant indirect risk by causing bacteraemia, contributing to, e.g., acute rejection.

### 6.1. Risk of Symptomatic UTI

All published studies analyzed the impact of ASB on the risk of symptomatic UTI development. In retrospective analyses, when all episodes of ASB were treated, ASB was a risk factor for the development of symptomatic UTI. Fiorante et al. showed that the incidence of acute graft pyelonephritis (AGPN) in KTx recipients was seven-fold higher in those with ASB compared with those without ASB. A history of recurrent ASB bacteriuria episodes (either two to five or more than five episodes) was a significant independent factor associated with AGPN [42]. The observations were similar in another retrospective study that included ASB as an independent risk factor for symptomatic UTIs. However, the authors indicated that only a small proportion of symptomatic UTIs were preceded by ASB with the same pathogen and concluded that recurrent ASB episodes may be considered a risk factor, a marker of increased susceptibility to symptomatic infections or a result of repeated and prolonged exposure to antibiotics [8]. When a comparison was made between cases of treated and untreated ASB, it clearly indicated that the risk of developing a symptomatic UTI or a symptomatic UTI requiring hospital admission increased only in those who received antibiotic therapy [43,44]. In pediatric KTx recipients, most untreated ASB episodes resolved without sequelae and on the other hand, most cases of both lower and upper symptomatic UTIs were de novo infections, which developed without preceding ASB [45].

Studies that assessed the prevalence, outcomes and management of asymptomatic bacteriuria (ASB) in KTx recipients are outlined in Table 1.

### 6.2. Impact on Kidney Allograft Function

A number of studies evaluated the impact of ASB on kidney allograft function. In a study on 189 KTx recipients who were followed for 36 months, which included 96 KTx recipients who developed 298 episodes of ASB, no differences were found in serum creatinine, creatinine clearance or proteinuria between patients with and without ASB [42]. In another retrospective analysis, the evolution of kidney allograft function also did not differ significantly between recipients with and without different UTI phenotypes, including ASB. However, patients suffering from symptomatic UTIs had significantly worse kidney allograft function measured by eGFR from the baseline in comparison with those without UTIs. The eGFR of recipients with a history of only ASB did not differ significantly when compared to both patients with symptomatic infections and patients with no infections [8]. Other studies have confirmed that both one-year patient and graft survival were comparable in recipients with or without ASB [11,44,46]. Furthermore, the antibiotic treatment of ASB did not affect the serum creatinine concentrations at baseline or the end of the study, when compared with the no treatment group [43,46,47,48,49].

### 6.3. Risk of Acute Rejection

A series of retrospective studies published in the literature have looked at the risk of acute rejection (AR) in KTx patients with ASB [8,40,42,44,46,50]. Fiorante et al. found that multiple ASB episodes were associated with rejection. However, the authors highlighted that not all the episodes of rejection were preceded by ASB episodes, so it was impossible to determine the causality of this relationship [42]. No other study has confirmed this finding regarding the risk for AR occurrence. Moreover, the antibiotic treatment of ASB had no effect on the risk of AR when compared to having no treatment [8,40,44,46,50].

**Table 1:** Studies that assessed the prevalence, outcomes and management of asymptomatic bacteriuria (ASB) in KTx recipients.

Study First Author, Year	Country	Group	Type of Study	Treatment	Results
Moradi, 2005 [47]	Iran	88 KTx recipients were randomized to either receive treatment for ASB (43 pts) episodes or not (45 pts). The mean participant age was 44.2 ± 12.7 years in the treatment arm and 40.9 ± 13.2 years in the control arm. There were 20 males in each arm.	A single-center RCT	Antibiotic in the treatment arm was chosen based on antimicrobial susceptibility testing and administered for 10 days.	The number of ASB episodes and symptomatic UTIs did not differ significantly between the groups (*p* > 0.05). Serum creatinine concentration did not increase significantly in either group up to the 12-month follow-up period (*p* > 0.05).
Fiorante, 2010 [42]	Spain	189 KTx recipients followed for 36 months, including 96 KTx recipients who developed 298 episodes of ASB. All received antibiotic treatment. Mean age of patients was 49 years and 39.68% were female.	A single-center retrospective study	Antibiotic in cases of ASB was chosen based on antimicrobial susceptibility testing and administered for 5–7 days.	No differences were found in serum creatinine, creatinine clearance or proteinuria between groups. The incidence of AGPN was 7-fold higher in patients with ASB.
El Amari, 2011 [51]	Switzerland	77 KTx recipients with 334 ASB episodes. Mean age of patients with ASB was 50 years and 58% were female. Cases were divided into four groups: Type I, high-grade bacteriuria with pyuria; Type II, high-grade bacteriuria without pyuria; Type III, low-grade bacteriuria with pyuria and Type IV, low-grade bacteriuria without pyuria. 30% of episodes were treated.	A single-center retrospective study	“*E. faecalis* bacteriuria was treated with either amoxicillin or amoxicillin–clavulanate. *E. coli* episodes were treated with ciprofloxacin, norfloxacin, amoxicillin, amoxicillin– clavulanate or fosfomycin, depending on the result of the antimicrobial susceptibility test.”	Persistent ASB developed in 45 (46%) treated episodes (2 Type I, 27 Type II, 8 Type III and 9 Type IV), with selection of resistant pathogen in 35 cases (78%). Spontaneous bacterial clearance occurred in 138 (59%) untreated episodes (15 Type I, 23 Type II, 9 Type III and 91 Type IV). Negative control cultures tended to be more frequent in treated Type I (*p* = 0.09) and in untreated Type II episodes (*p* = 0.08).
Gołębiewska, 2012 [8]	Poland	209 KTx recipients within 1 year post-KTx, including 83 KTx recipients who developed 170 ASB episodes. All episodes were treated. Mean age of patients was 46.38 ± 14.05 years and 40.7% were female.	A single-center retrospective study	Antibiotic was chosen based on antimicrobial susceptibility testing	The following risk factors for developing both ASB and symptomatic UTIs were identified: female sex, use of induction with anti-thymocyte globulin, comorbidity measured by Charlson Comorbidity Index, history of AR and CMV infection. ASB was an independent risk factor for symptomatic UTIs, but only 21 of 152 episodes of symptomatic UTIs were preceded by ASB with the same pathogen.
Green, 2013 [43]	Israel	112 KTx recipients within 1 year post-KTx. 22 patients received antibiotic treatment (19.6%) for ASB episodes, while 90 patients did not. The mean participant age was 52.7 ± 12.6 in the treated ASB group and 49.2 ± 16.2 in the untreated group, and the groups were 68% and 64% female, respectively.	A single-center retrospective study	In treated cases the antibiotic was chosen based on antimicrobial susceptibility testing and given for at least 3 days.	A composite endpoint of hospitalization for symptomatic UTI or more than 25% reduction in the eGFR 30 days after the documentation of ASB occurred in 4/22 (18.2%) of the treated patients versus 5/90 (5.6%) of the untreated patients [OR = 3.78, 95% CI 0.9–15]. The risk of developing symptomatic UTI after ASB was almost 3-fold higher (*p* < 0.05) and the total number of hospitalization days at 6-months post-ASB was also significantly higher (*p* < 0.026) in the treated group.
Arencibia, 2016 [52]	Spain	20 untreated ASB cases, 28 treated ASB cases, and 13 treated symptomatic UTIs. Mean participant age was 56 ± 13.5 in cases of positive cultures collected in the summer months and 53 ± 15.6 in cases of positive cultures collected in the winter months. The groups were 51.8% and 55% female, respectively.	A single-center prospective observational study	The decision to treat the ASB and the choice of the antimicrobial therapy was at the discretion of the treating physician.	Only 10 patients required hospitalization during follow-up, and all of them belonged to the treated ASB/symptomatic UTI group. Bacterial clearance after the treatment occurred in 20/41 treated patients (48.9%) and spontaneously in 14/20 patients with untreated ASB (70%). In the treated group, 47.6% developed a new resistance to another antibiotic.
Origuen, 2016 [48]	Spain	112 KTx recipients with at least one ASB episode between 2 and 24 months post-KTx randomized (1:1 ratio) to the treatment group (systematic antibiotic treatment for all episodes of ASB (53 patients)) or control group (no antibiotic therapy (59 patients)). The mean participant age was 55.4 ± 14.5 in the treatment arm and 53.04 ± 15.8 in the control arm. There were 28 and 31 males, respectively. Systematic screening for ASB was similar in both groups. The primary outcome was the occurrence of AGPN at 24-months follow-up. Secondary outcomes included lower UTI, AR, Clostridium difficile infection, colonization or infection by MDR bacteria, graft function and all-cause mortality.	A single-center RCT	Antibiotic in the treatment arm was chosen based on antimicrobial susceptibility testing.	No differences in the primary outcome in the intention-to-treat population (7.5% (4 of 53) in the treatment group vs. 8.4% (5 of 59) in the control group; or the per-protocol population (3.8% (1 of 26) in the treatment group vs. 8.0% (4 of 50) in the control group). Secondary outcomes: no differences.
Singh, 2016 [53]	Netherlands	343 KTx recipients, including 212 (61.8%) who received TMP-SMX as PJP prophylaxis. 63 (18.4%) developed ASB without UTI, 26 (7.6%) developed cystitis and 43 (12.5%) developed AGPN. Mean age of patients with ASB was 52 years and 44.3% were female.	A single-center retrospective study	N/A	TMP-SMX as PJP prophylaxis was not associated with reduced prevalence of ASB (HR = 1.52, 95% CI = 0.79–2.94, *p* = 0.213), reduced incidence of cystitis (HR = 2.21, 95% CI = 0.76–6.39, *p* = 0.144) or AGPN (HR = 1.12, 95% CI = 0.57–2.21, *p* = 0.751).
Goh, 2017 [44]	Singapore	171 adult KTx recipients, in whom protocol urine cultures were taken at 1 month post-transplantation. The mean age of participants was 47.3 ± 13.7 years and 50.9% were male. Forty-one (24%) KTx recipients had ASB at 30 days post-transplant. All episodes were treated.	A single-center retrospective study	Antibiotic in cases of ASB was chosen based on antimicrobial susceptibility testing and administered for 14 days.	MDR organisms accounted for 43.9% of infections. Female sex and deceased donor recipients were independent predictors of 30-day bacteriuria, which predicts subsequent hospitalization for symptomatic UTI. One-year patient and graft survival were similar in recipients with or without ASB.
Kotagiri, 2017 [40]	Australia	276 KTx recipients within 1 year post-KTx. 158 (57%) had no bacteriuria, 75 (27%) had ASB, 21 (8%) had symptomatic lower UTIs, and 22 (8%) had upper UTIs. The mean age of participants was 51 ± 13 years and 67% were male.	A two-center retrospective study	The decision whether to treat ASB episode was at the discretion of the treating physician	Female gender was a risk factor for infection (*p* = 0.002), and the use of double-J ureteral stent was a risk factor for both ASB and symptomatic UTIs (*p* = 0.003). The likelihood of symptomatic UTI in the same organism was significantly higher (*p* = 0.002) after untreated ASB (*n* = 185) than in cases of treated ASB (*n* = 139).
Bonneric, 2019 [45]	France	37 pediatric KTx recipients with 171 ASB episodes. Primary outcome was the cumulative incidence of AGPN or LUTI occurring between 2 and 24 months post-KTx. The secondary outcomes were AGPN or LUTI due to MDR bacteria, long-term GFR evolution and graft loss. The mean age of participants was 11.2 ± 5.2 years and 59% were male.	A single-center retrospective study	No information provided on the treatment of ASB in the treated cases.	One hundred and sixty-four ASB episodes were untreated (95.9%) and 150 episodes (91.5%) were not followed by a symptomatic UTI. Ten episodes (6.1%) led to AGPN and 4 (2.4%) led to LUTI. There were 53 episodes of AGPN: 10 (18.9%) after untreated ASB and 43 (81.1%) de novo. There were 11 episodes of LUTI: 4 (36.4%) after untreated ASB and 7 (63.6%) de novo. There was no graft loss as a result of UTI.
Coussement, 2019 [10]	Belgium/France	500 consecutive KTx recipients ≥ 2 months post-KTx were screened for ASB	A multicenter cross-sectional study	N/A	The prevalence of ASB was 3.4% (17/500 patients). ASB was significantly associated with female gender (risk ratio 3.7, 95% CI 1.3–10.3, *p* = 0.007) and older age (mean age of bacteriuric patients: 61 ± 12 years, versus 53 ± 15 years for non-bacteriuric patients, *p* = 0.03).
Sabe, 2019 [54]	Spain	205 KTx recipients. ASB occurred in 41 (42.3%) and 46 (50.5%) patients in the treatment and no treatment groups, respectively. The mean participant age was 61.048 ± 11.582 years in the treatment arm and 60.194 ± 11.412 years in the control arm. There were 36.6% and 45.7% males, respectively.	A multicenter RCT	Antibiotic in the treatment arm was chosen based on antimicrobial susceptibility testing and administered for 5–7 days. If fosfomycin was used, the treatment lasted 1–3 days.	There were no differences in the occurrence of AGPN in the intention-to-treat population (12.2% (5 of 41) in the treatment group vs. 8.7% (4 of 46) in the no treatment group; RR, 1.40; 95% CI, 0.40–4.87) or the per-protocol population (13.8% (4 of 29) in the treatment group vs. 6.7% (3 of 45) in the no treatment group; RR, 2.07, 95% CI, 0.50–8.58). No differences were found in bacteremic AGPN, cystitis, AR, graft function, graft loss, opportunistic infections, need for hospitalization and mortality. Antibiotic resistance was increased in the treatment arm.
Brune, 2021 [11]	Switzerland	2368 KTx recipients with 2363 UTI events within the first year after KTx. Patients were divided into four groups: (i) no colonization or UTI (*n* = 1404; 59%), (ii) colonization/ASB only (*n* = 353; 15%), (iii) occasional UTI with 1–2 episodes (*n* = 456; 19%), and (iv) recurrent UTI with ≥3 episodes (*n* = 155; 7%). The mean age of participants in the 4 groups was 54, 54, 57 and 58 years, respectively. The proportion of females was 27%, 39%, 52% and 59%, respectively	A multicenter prospective observational study	ASB cases were treated in 2/6 participating centers within the first 6 months after KTx and until double J-stent removal.	No differences in one-year mortality, graft loss rate and long-term patient survival among the four groups
Coussement, 2021 [49]	Belgium/France	199 KTx recipients ≥2 months post-KTx were randomly assigned to antibiotics for the treatment of ASB (100 pts) or no therapy (99 pts). The mean participant age was 60.1 ± 11.6 years in the no treatment arm and 60.2 ± 11.5 years in the treatment arm. There were 74 and 77 females, respectively.	A multicenter RCT	The choice of the antimicrobial therapy was at the discretion of the treating physician based on antimicrobial susceptibility testing and the treatment was administered for 10 days.	The number of symptomatic UTIs did not differ between the antibiotic and no treatment groups (27%, 27/100 versus 31%, 31/99).
Fontsere, 2021 [46]	Spain	197 KTx recipients: 175 (88.8%) with ASB and 22 (11.2%) with cystitis. The decision to treat the ASB and the choice of the antimicrobial therapy was at the discretion of the treating physician. The median age of participants was 59 years and 52.8% were female.	A single-center prospective observational study	The decision to treat the ASB and the choice of the antimicrobial therapy was at the discretion of the treating physician.	The treatment of ASB increased the rates of microbiologic relapses and reinfections; treated ASB patients showed a trend towards developing symptomatic urinary tract infections in the following 6 months.
Ruiz-Ruigomez, 2021 [55]	Spain	This study assessed the rate of microbiological eradication and factors predicting microbiological failure in 137 episodes of ASB in 133 KTx recipients treated with oral fosfomycin. The mean age of participants at KTx was 54.8 ± 13.6 years and 57.1% were female.	A multicenter retrospective analysis	Oral fosfomycin administered for 2–9 days.	Rate of microbiological failure at month 1 was 40.1% (95% CI, 31.9% to 48.9%) for the whole cohort and 42.3% (95% CI, 31.2% to 54.0%) for episodes due to MDR pathogens. The predictors of microbiological failure were: previous UTI (OR, 2.42; 95% CI, 1.11 to 5.29; *p* value = 0.027) and use of fosfomycin as salvage therapy (OR, 8.31; 95% CI, 1.67 to 41.35; *p* value = 0.010). There were no severe adverse events related to the treatment.

AGPN—acute graft pyelonephritis, AR—acute rejection, ASB—asymptomatic bacteriuria, CI—confidence interval, CMV—cytomegalovirus, HR—hazard ratio, KTx—kidney transplant, LUTI—lower urinary tract infection, MDR—multidrug resistant, N/A—not applicable, pts—patients, OR—odds ratio, PJP—Pneumocystis jiroveci pneumonia, RCT—randomized controlled trial, RR—relative risk, TMP-SMX—trimethoprim sulfametoxazole, UTI—urinary tract infection.

## 7. Management

The process of screening for and treating ASB is a common practice in many European kidney transplantation centers [56,57]. Seventy-two percent of European physicians involved in the care of KTx recipients always screen for ASB in their patients, while only 6% never treat ASB [57]. We fear that infections can rapidly progress in immunosuppressed patients and that it may be particularly difficult to distinguish between ASB and a true UTI in the setting of subtle symptoms mitigated by immunosuppression. Even in the general population, where guidelines are clear-cut, antimicrobials are often unnecessarily used to treat cases of ASB, especially in patients with poor functional status and men [58].

On the other hand, the concern for unnecessary treatment of ASB originates from the rapidly growing bacterial resistance and the perception of antibiotic stewardship as a public health priority [59]. UTIs caused by multidrug resistant Gram-negative bacteria are more and more common, especially in the population of solid organ transplant recipients, and are a growing concern due to limited treatment options [60,61].

On an individual level, the unnecessary exposure to antibiotics may be associated with potential side effects, negatively impacting one’s microflora, and, therefore, leading to undesirable consequences such as increasing the risk of colonization or infection by multidrug-resistant bacteria, such as *Clostridioides difficile* infection, etc.

Whether antibiotic treatment of ASB is in fact helpful or harmful in preventing symptomatic infections in KTx recipients has not been fully elucidated so far. In a study by Cai et al. of a non-transplant population of young women, the treatment of ASB in patients affected by recurrent UTIs resulted in a higher rate of symptomatic UTIs [62]. There is a growing body of evidence from various types of studies showing that screening for and treating ASB is also not beneficial in most KTx recipients. Earlier retrospective studies showed that the incidence of symptomatic UTIs, including AGPN, was higher in KTx recipients with multiple, recurring episodes of ASB, either despite or because of the implemented antibiotic treatment [8,32,44]. At the same time, very few symptomatic UTIs were preceded by ASB with the same causative agent [8]. In a study by Green et al., the use of antibiotics in cases of ASB increased the risk of both symptomatic UTI and a hospital stay [43]. Interestingly, the chance of bacterial clearance was higher in KTx recipients with ASB who received no treatment when compared to those who were administered antibiotics [52]. The findings were similar in pediatric KTx recipients—91.5% of untreated cases of ASB were not followed by a symptomatic UTI and over 80% of cases of AGPN were not preceded by ASB [45]. Only a single study of 185 KTx recipients showed that untreated ASB was significantly more likely to be followed by a subsequent episode of symptomatic UTI in the same organism in comparison to cases of treated ASB [40]. In a recently published study by Ruiz-Ruigomez, which assessed the rate of microbiological eradication after treatment of ASB episodes with fosfomycin, the treatment failure rate exceeded 40% [55]. Moreover, the routine use of trimethoprim-sulfametoxazole prophylaxis did not reduce the prevalence of ASB [53].

To date, the results of four randomized controlled trials investigating the use of antibiotics for the treatment of ASB in KTx recipients have been published [47,48,49,54]. The results were consistent and showed no apparent benefit of systematic screening and treatment of ASB in the populations of patients beyond 2 months post-transplant. The use of antibiotics did not decrease the likelihood of developing a symptomatic UTI, but promoted the emergence of resistant organisms.

Interestingly, in a group of 420 KTx recipients, the isolates from preoperative urine, preoperative urethral swab and postoperative urinary catheter tip cultures also showed poor concordance with the causal organism during an early symptomatic UTI. Thus, perioperative microbiological screening had very limited or no value [63].

As most studies have failed to demonstrate any benefit of the “screen and treat strategy” in terms of incidence of symptomatic UTIs or long-term kidney allograft function, a paradigm shift toward a more conservative approach has occurred in the management of post-transplant ASB. Indeed, most of the more recent guidelines issued by, e.g., the Spanish Society of Clinical Microbiology and Infectious Diseases (SEIMC), the Infectious Diseases Society of America (IDSA), the American Society of Transplantation Infectious Diseases Community of Practice and the European Association of Urology have clearly recommended against screening for or treating ASB beyond the first month post-transplant (Table 2) [13,64,65,66]. Unfortunately, a survey conducted among European transplant nephrologists involved in the care of adult KTx recipients indicated a huge discrepancy between existing guidelines and everyday clinical practice, with most physicians screening for and treating ASB in their patients, even beyond the second month post-KTx [57].

The proportion of different clinical manifestations of UTIs differs between early and late periods after KTx, and ASB episodes predominate in the first post-transplant month [8]. However, evidence concerning ASB occurring during the first post-transplant month or in KTx recipients with indwelling urinary catheters is missing, since these episodes were excluded from all published randomized clinical trials [47,48,49,54]. The net state of immunosuppression is highest during the first post-transplant months and decreases thereafter. The presence of indwelling urinary catheters and double-J stents and the highest rate of urologic interventions during this period may increase the risk of progression from ASB to symptomatic UTI, especially given the fact that symptoms of urinary tract infection may be impeded by graft denervation and inflammatory signs may be altered by immunosuppressive medications. Given the abovementioned differences, the recommendation to not screen for and treat ASB may not be applicable during months 1–2 post-transplant. Further studies are needed to evaluate the efficacy and safety of the “screen and treat strategy” in the early post-KTx period.

## 8. Conclusions

ASB is a common finding in KTx recipients, especially females. Its etiology mirrors the etiology of symptomatic UTIs, with a predominance of Gram-negative rods typically colonizing the gastrointestinal tract. It seems that ASB strains are able to prevent the development of the host’s systemic inflammatory reaction, although they may still induce a local inflammatory response of unrecognized significance. Given that there is no rigorous molecular definition to differentiate between uropathogenic or merely urocolonizing strains, and that KTx recipients are immunocompromised and suffer from numerous urological malformations (vesicoureteral reflux is a permanent symptom after KTx, and KTx recipients are exposed to invasive diagnostic and therapeutic procedures involving the urinary tract, predisposing them to the rapid development of symptomatic, potentially severe infections), it is common practice to screen for and treat ASB episodes. However, there is a growing body of evidence from clinical trials that this strategy is not beneficial in patients more than 2 months post-transplant in terms of symptomatic UTI prevention. In addition, antibiotic treatment of ASB increases antibiotic use and, therefore, promotes the emergence of more resistant strains. As antibiotics for ASB are typically prescribed as a consequence of the positive result of a urine culture, efforts should be made to stop obtaining urine cultures in KTx recipients who have no signs or symptoms of UTI. There is also no clear-cut repercussion of ASB on kidney allograft function prognosis; thus, there is no rationale to support antibiotic use in ASB episodes in patients more than 2 months post-transplant.

Despite its frequency, ASB after KTx is still an understudied phenomenon, especially in recipients who are 1–2 months post-transplant or who have an indwelling urinary catheter. Further randomized studies are required to provide evidence on how a screen-and-treat strategy for ASB would affect clinical outcomes in the early period post-KTx.

## Figures and Tables

**Table 2:** Published guidelines on management of asymptomatic bacteriuria (ASB) in KTx recipients.

Guideline, Year	Recommendations
KDIGO Clinical practice guideline for the care of kidney transplant recipient, 2009 [67]	No recommendations on ASB treatment and screening in KTx recipients
Guidelines of the Spanish Society of Clinical Microbiology and Infectious Diseases (SEIMC), 2017 [64]	“For kidney transplant patients, the screening and treatment of ASB is only recommended in the first month after transplantation (B-III). For cases of hematopoietic stem cell transplants and SOTs other than kidney transplants, no recommendations for the screening and treatment of ASB can be made (C-III). Systemic antifungal therapy for asymptomatic candiduria is not recommended for transplant patients, except for neutropenic patients or those scheduled to undergo urological procedures (D-III)”.
The Japanese Association for Infectious Disease/Japanese Society of Chemotherapy (JAID/JSC) Guide/Guidelines to Clinical Management of Infectious Disease, 2017 [68]	No recommendations on ASB treatment and screening in KTx recipients
National Institute for Health and Care Excellence Guideline on Antibiotic prescribing in lower urinary tract infection, 2018 [69]	No recommendations on ASB treatment and screening in KTx recipients
Urinary Tract Infections in Solid Organ Transplant Recipients: Guidelines from the American Society of Transplantation Infectious Diseases Community of Practice, 2019 [65]	“We recommend against routinely collecting urine cultures or treating bacteriuria in asymptomatic KTx recipients more than 2 months after KTx (Strong, Moderate). If screening asymptomatic KT recipients any time in the posttransplant period and ASB is found, a second urine culture (minimizing risk of contamination) should be collected and reviewed prior to decision about whether or not to treat ASB. We strongly recommend observation without treatment of asymptomatic KT recipients who show clearance of the initial bacteriuria or development of different organism in the urine (Strong, Moderate). Because of the uncommon occurrence of asymptomatic pyelonephritis, treatment of persistent ASB can be considered in patients with an associated unexplained rise in creatinine (Weak, Low). Multidrug resistant ASB should not be treated. The risks of inducing further antibiotic resistance outweigh any potential theoretical benefit of treating ASB (Strong, Moderate)”.
Clinical Practice Guideline for the Management of Asymptomatic Bacteriuria: 2019 Update by the Infectious Diseases Society of America, 2019 [13]	“In renal transplant recipients who have had renal transplant surgery >1 month prior, we recommend against screening for or treating ASB (strong recommendation, high-quality evidence). Remarks: There is insufficient evidence to inform a recommendation for or against screening or treatment of ASB within the first month following renal transplantation”.
European Association of Urology (EAU) Guidelines On Urological Infections, 2021 [66]	“Do not screen or treat asymptomatic bacteriuria in patients with renal transplants. (Strong)”

ASB—asymptomatic bacteriuria, KTx—kidney transplant.

## Data Availability

Not applicable.
